# Ataxia, dystonia and myoclonus in adult patients with Niemann-Pick type C

**DOI:** 10.1186/s13023-016-0502-3

**Published:** 2016-09-01

**Authors:** L. H. Koens, A. Kuiper, M. A. Coenen, J. W. J. Elting, J. J. de Vries, M. Engelen, J. H. T. M. Koelman, F. J. van Spronsen, J. M. Spikman, T. J. de Koning, M. A. J. Tijssen

**Affiliations:** 1 Department of Neurology, University of Groningen, University Medical Center Groningen, Hanzeplein 1, 9700 RB Groningen, The Netherlands; 2 Department of Clinical Neuropsychology, University of Groningen, University Medical Center Groningen, Hanzeplein 1, 9700 RB Groningen, The Netherlands; 3 Department of Neurology, University of Amsterdam, Academic Medical Center, Meibergdreef 9, 1105 AZ Amsterdam, The Netherlands; 4 Division of Metabolic Diseases, University of Groningen, University Medical Center Groningen, Beatrix Children’s Hospital, Hanzeplein 1, 9700 RB Groningen, The Netherlands; 5 Department of Clinical and Developmental Neuropsychology, University of Groningen, Faculty of Behavioral and Social Sciences, Grote Kruisstraat 2/1, 9712 TS Groningen, The Netherlands; 6 Department of Genetics, University of Groningen, University Medical Center Groningen, Hanzeplein 1, 9700 RB Groningen, The Netherlands

**Keywords:** Niemann-Pick type C, Cortical myoclonus, EEG-EMG coherence, Ataxia, Cognitive deficits

## Abstract

**Background:**

Niemann-Pick type C (NP-C) is a rare autosomal recessive progressive neurodegenerative disorder caused by mutations in the NP-C 1 or 2 gene. Besides visceral symptoms, presentation in adolescent and adult onset variants is often with neurological symptoms. The most frequently reported presenting symptoms of NP-C in adulthood are psychiatric symptoms (38 %), cognitive decline (23 %) and ataxia (20 %). Myoclonus can be present, but its value in early diagnosis and the evolving clinical phenotype in NP-C is unclear. In this paper we present eight Dutch cases of NP-C of whom five with myoclonus.

**Methods:**

Eight patients with genetically confirmed NP-C were recruited from two Dutch University Medical Centers. A structured interview and neuropsychological tests (for working and verbal memory, attention and emotion recognition) were performed. Movement disorders were assessed using a standardized video protocol. Quality of life was evaluated by questionnaires (Rand-36, SIP-68, HAQ). In four of the five patients with myoclonic jerks simultaneous EEG with EMG was performed.

**Results:**

A movement disorder was the initial neurological symptom in six patients: three with myoclonus and three with ataxia. Two others presented with psychosis. Four experienced cognitive deficits early in the course of the disease. Patients showed cognitive deficits in all investigated domains. Five patients showed myoclonic jerks, including negative myoclonus. In all registered patients EEG-EMG coherence analysis and/or back-averaging proved a cortical origin of myoclonus. Patients with more severe movement disorders experienced significantly more physical disabilities.

**Conclusions:**

Presenting neurological symptoms of NP-C include movement disorders, psychosis and cognitive deficits. At current neurological examination movement disorders were seen in all patients. The incidence of myoclonus in our cohort was considerably higher (63 %) than in previous publications and it was the presenting symptom in 38 %. A cortical origin of myoclonus was demonstrated. Our data suggest that myoclonus may be overlooked in patients with NP-C. All patients scored significantly lower on physical domains of HRQoL. Symptomatic treatment of movement disorders may improve physical functioning and subsequently HRQoL.

## Background

Niemann-Pick type C (NP-C) is a rare autosomal recessive neurodegenerative disorder with an estimated incidence of 1 per 120.000 live births [1]. The disorder is caused by mutations in the NP-C 1 (95 %) or NP-C 2 gene (5 %). Although the exact cellular functions of the proteins remain to be elucidated, they are involved in cellular lipid transport/trafficking and mutated proteins lead to (lysosomal) accumulation of lipids. Lipid storage products accumulate in many organs including the brain, resulting in cerebral degeneration with progressive neurological symptoms, cognitive decline and psychiatric symptoms. NP-C is a very heterogeneous disorder with regard to age of onset and clinical presentation. The age of onset can vary from the newborn period to adulthood. The neonatal variant usually presents with liver disease at or soon after birth and causes early death. After the newborn period, early- and late- infantile NP-C (0–6 years), juvenile NP-C (6–15 years) and adolescent/adult NP-C (>15 years) can occur and patients mainly present with neurological symptoms, although visceral symptoms, in particular in children also occur [2, 3].

The neurological symptoms in the adolescent or adulthood form of NP-C start insidiously and have a slower progression compared to children [2]. The adult onset forms of NP-C were considered rare, but recent studies suggest that NP-C in adulthood is under-recognized [2–4]. Long delays between the onset of neurological manifestations and genetic conformation of NP-C are not uncommon, [3] likely because of the more insidious course and nonspecific presenting symptoms. The most frequently reported presenting symptoms of NP-C in adulthood are psychiatric symptoms (38 %), cognitive decline (23 %), ataxia (20 %) and other movement disorders including dystonia (11 %) [4, 5]. Especially the combination of neurological, visceral and psychiatric symptoms is considered to be highly suggestive for the diagnosis NP-C [6]. As treatment options for NP-C have become available, early identification of NP-C is important to prevent further deterioration and progress of neurological manifestations [7].

The frequency of myoclonus in NP-C was found around 8, 5 % in a retrospective review, [8] but the frequency of myoclonus being a presenting symptom is unknown. Canafoglia et al. described a single case of late-infantile NP-C with severely disabling cortical myoclonus at the age of eight years. They concluded that high-frequency myoclonus may be subtle and easily overlooked at disease onset, potentially leading to considerable delays in diagnosis [9]. Based on our clinical experience in other patients with neurogenetic disorders with multiple movement disorders, [10] myoclonus can be difficult to identify indeed, especially when ataxia is present in the same patient. For this reason we suspect that myoclonus is under-recognized in NP-C.

Psychiatric disorders and cognitive deficits are common in adults with NP-C and can precede the motor symptoms [5]. Psychosis is one of the characteristic disorders in the adult onset form of NP-C, but patients can present with a variety of unspecific psychiatric problems. Hence, the diagnosis is often delayed [11] and treatment [12] can be delayed for several years. Vanier et al. described depression and behavioral problems with aggression and withdrawal as possible psychiatric symptoms that precede the motor signs of NP-C [1]. The number of studies on cognitive functioning of patients with NP-C is limited, but the existing studies show deficits in language, information processing speed and divided attention, memory, visuospatial skills and constructional praxis [13, 14]. Deficits in social cognition and other domains contribute to the observed cognitive decline that can be one of the precursors of NP-C [8].

In this paper we present eight Dutch cases of NP-C. Early symptoms of NP-C include movement disorders, psychiatric symptoms and cognitive deficits. For this reason we investigated neuropsychological functioning in seven patients with NP-C. We aim to increase knowledge of movement disorders and neuropsychiatric functioning in NP-C. The goal of our study was to determine the frequency of myoclonus in NP-C patients and to determine the anatomical origin of the myoclonus. We considered that recognition of myoclonus might be an important diagnostic sign for NP-C and enables symptomatic treatment to improve overall functioning [10].

## Methods

We assessed the type and severity of neurological symptoms/movement disorders, behavioral and cognitive deficits, health related quality of life (HRQoL) and daily functioning in patients with NP-C. Inclusion criteria were a confirmed molecular diagnosis of NP-C and age at examination above 18 years. Twelve patients fulfilling the inclusion criteria were found. Two patients passed away during recruitment, two patients did not consent to participate for various reasons, such as end stage disease. Finally, eight patients were included from two Dutch University Medical Centers (University Medical Center Groningen and Academic Medical Center Amsterdam). This study was approved by the medical ethical committee of the University Medical Center Groningen (METc2013.417). Data on demographics, age of diagnosis, symptoms at onset, disease characteristics and treatment were collected. To determine the presence and severity of a movement disorder, all patients underwent a standardized videotaped neurological examination. These videos were scored by an expert panel through consensus discussions and by using the Global Clinical Impressions Scale (GCI) [15]. In those patients who were able to complete the tests and questionnaires, extensive neuropsychological testing was performed. The assessment took place varying from directly after the diagnosis to 4 years after diagnosis. The neuropsychological assessment covered emotion recognition as part of social cognition, [16] but also intelligence, [17–19] working memory, [20] verbal memory, [21] attention, [22] and verbal fluency [19]. Results of the neuropsychological assessment were compared to normative data as mentioned in the test manuals. HRQoL and daily functioning were examined using questionnaires (36-Item short form health survey (Rand-36), Dutch Health Assessment Questionnaire (HAQ) and Sickness Impact Profile 68 (SIP-68)). The scores on HRQoL and daily functioning in our cohort were compared with published reference ranges for the Dutch population [23, 24]. Also correlations between the (non-) motor symptoms and QoL and daily functioning were studied.

If patients were found to have myoclonic jerks at neurological examination, additional neurophysiological tests were performed to analyze the relationship between myoclonus and cortical events. These tests consisted of simultaneous EEG and polymyography. Cortical activities were recorded by standard EEG according to the international 10–20 system. Muscle activity was measured by nine channel surface EMG with the electrodes placed on extensor and flexor muscles of both upper and forearms, and on the abductor pollicis brevis muscle on one side. A standard myoclonus protocol was performed, assessing rest periods, several postures and also the effect of several stimuli, such as auditory, tactile and lightflash stimuli. Back-averaging and coherence analysis were performed to relate the cortical signals to EMG burst.

## Results

Eight patients with a confirmed diagnosis of Niemann-Pick type C were included. Their ages ranged from 19 to 61 years. Five patients presented with juvenile onset NP-C and three patients with adolescent/adult onset NP-C. All patients had mutations in the NP-C 1 gene. In seven patients the mutations were detected through direct Sanger sequencing of the NP-C genes and in one patient (nr. 3) the diagnosis was made through a gene panel with 88 genes related to movement disorders. In six patients Filipin staining was performed. In patient 1, 4, 5, 6, 7 and 8 Filipin stain was positive. No Filipin stain was performed in patient 2 and 3.

The characteristics of the patients are shown in Table 1.

**Table 1:** Patient characteristics

Patient	Sex	Mutation	Age at neurological symptoms (yrs)	Age at diagnosis (yrs)	Age at examination (yrs)	Miglustat
1	Female	Compound heterozygous mutations c.1211G > A (p. Arg404Gln) and c.2861C > T (p. Ser954Leu)	18	23	35	No
2	Female	Homozygous mutations c.3182 T > C (p.IIe1061Thr)	8	21	25	Yes
3	Male	Compound heterozygous mutations c.2474A > G (p.Tyr825Cys) and c.3019C > G (p.Pro1007Ala)	52	59	61	Yes
4	Female	Homozygous mutations p.Gly640Arg	14	18	20	Yes
5	Male	Compound heterozygous mutations c.346C > T (p.R116X) and c.247A > G (p. Y825C)	14	17	19	No
6	Male	Compound heterozygous mutations c.1918G > A (p.Gly640Arg) and c.3451G > A (p.Ala1151Thr)	6	14	19	Yes
7	Male	Compound heterozygous mutations c.3203C > T (p.Thr1068IIe) and c.3614C > A (p.Thr1205Lys)	40	52	58	Yes
8	Female	Homozygous mutations c.3182 T > C (p.IIe1061Thr)	11	6	26	No

Three patients (nr. 2, 4, 8) had a history of prolonged neonatal jaundice. Two other patients (nr. 5, 6) presented with visceral signs later in childhood including (hepato) splenomegaly. In patient number 8 jaundice and hepatosplenomegaly were reason for additional investigations which led to the diagnosis of NP- C before any other symptom did occur.

In five out of eight patients non-specific changes were seen on brain MRI, including white matter changes and/or global (including cerebellar) atrophy.

### Presenting neurological symptoms and current neurological abnormalities

In retrospect, when asked patients or parents, involuntary movements were the first noticed neurological symptom in six patients. Three presented with jerks very suggestive of myoclonus (age 20, 25, 61) and three with signs of ataxia (age 19, 26, 58). Psychosis was presenting symptom in two patients, but neurological examination revealed a movement disorder as well in both patients at the initial presentation. In patient number 1 there were signs of gait ataxia and VSGP (vertical supranuclear gaze palsy), in patient number 5 dystonia of the right arm and VSGP were present.

In half of the patients signs suggestive of VSGP (impaired downward gaze manifesting itself with troubles playing piano or working on the computer) and cognitive problems were mentioned also early in disease by patients or parents. However, unlike movement disorders or psychosis, none of the patients sought medical attention for this (Table 2).

**Table 2:** Presenting and current movement disorders

Patient	Presenting movement disorder	Severity of current movement disorder (GCI) ^a^				
		Ataxia	Myoclonus	Dystonia	Tic	Overall severity
1	Ataxia	5	1	3	1	5
2	Myoclonus	3	4	1	1	4
3	Myoclonus	2	3	1	1	3
4	Myoclonus	4	5	3	1	5
5	Dystonia	1	1	2	1	2
6	Ataxia	4	1	4	1	4
7	Ataxia	5	4	3	1	5
8	Ataxia	4	2	1	2	4

^a^ *GCI* Global Clinical Impressions Scale, 1 (no movement disorder) - 7 (among the most extremely affected patients)

In all patients the presenting movement disorder was currently also the predominant movement disorder seen. However, seven patients developed additional movement disorders. Four of them had one additional movement disorder and the other three patients two. This showed that the majority of patients had mixed and sometimes complex movement disorder phenotypes.

Upon current neurological examination, myoclonic jerks were found to be present in five patients. In one patient (nr. 3) the jerks were generalized and present in all extremities, trunk, face and tongue. In the other four patients, myoclonus was predominantly located distally in the limbs and aggravated with posture maintenance and active movements. The upper extremities were usually more affected than the lower extremities. In one of these patients (nr. 4) there were perioral myoclonic jerks as well. In all patients but one (nr. 3) myoclonus was stimulus-sensitive. Negative myoclonus was seen in all patients. On the GCI the myoclonus was considered to be significant in two patients (4 and 5 on the scale of 7). Myoclonus was however not the most frequently observed movement disorder. Seven patients developed ataxia of the extremities in the course of the disease, including three patients who presented with myoclonus (nr. 2, 3, 4).

Dystonia was also present in five patients, with only in one of them dystonia being the presenting symptom. In two patients generalized dystonia was observed; one patient (nr. 1) suffered from dystonia of face, trunk and extremities and in another patient (nr. 4) facial and cervical dystonia were present combined with dystonic posturing of the feet. In the other three patients dystonia was more confined to the neck and shoulder region. Patient 5 showed minimal dystonia of the right arm and patient 6 had a combination of cervical and upper extremity dystonia. Finally, patient 7 had severe cervical dystonia with also dystonic postures of the left hand while writing.

One patient showed a tic of the head. Tremor, chorea and parkinsonism were not observed in our cohort.

### Myoclonus and electrophysiological characteristics

In four out of five patients (nr. 2, 3, 4, 7) with myoclonus, EEG-EMG polygraphic study was performed. Patients were subjected to a standard myoclonus protocol. Table 3 shows the results of the neurophysiological analysis of the four tested patients.

**Table 3:** Electrophysiological characteristics

Patient	Duration	Frequency	Location	Type	Back-averaging	Coherence	Reflex myoclonus
2	40–60 ms	0.1-10Hz	Distal	Positive and negative	Positive	Positive	No
3	50–100 ms	0.1-10Hz	Distal	Positive and negative	Positive	Positive	No
4	40–70 ms	0.1-10Hz	Distal	Positive and negative	NA^a^	Positive	No
7	<70 ms	0.1-10Hz	Proximal and distal	Positive and negative	NA^a^	Positive	No

^a^ *NA* not applicable

**Fig. 1:**
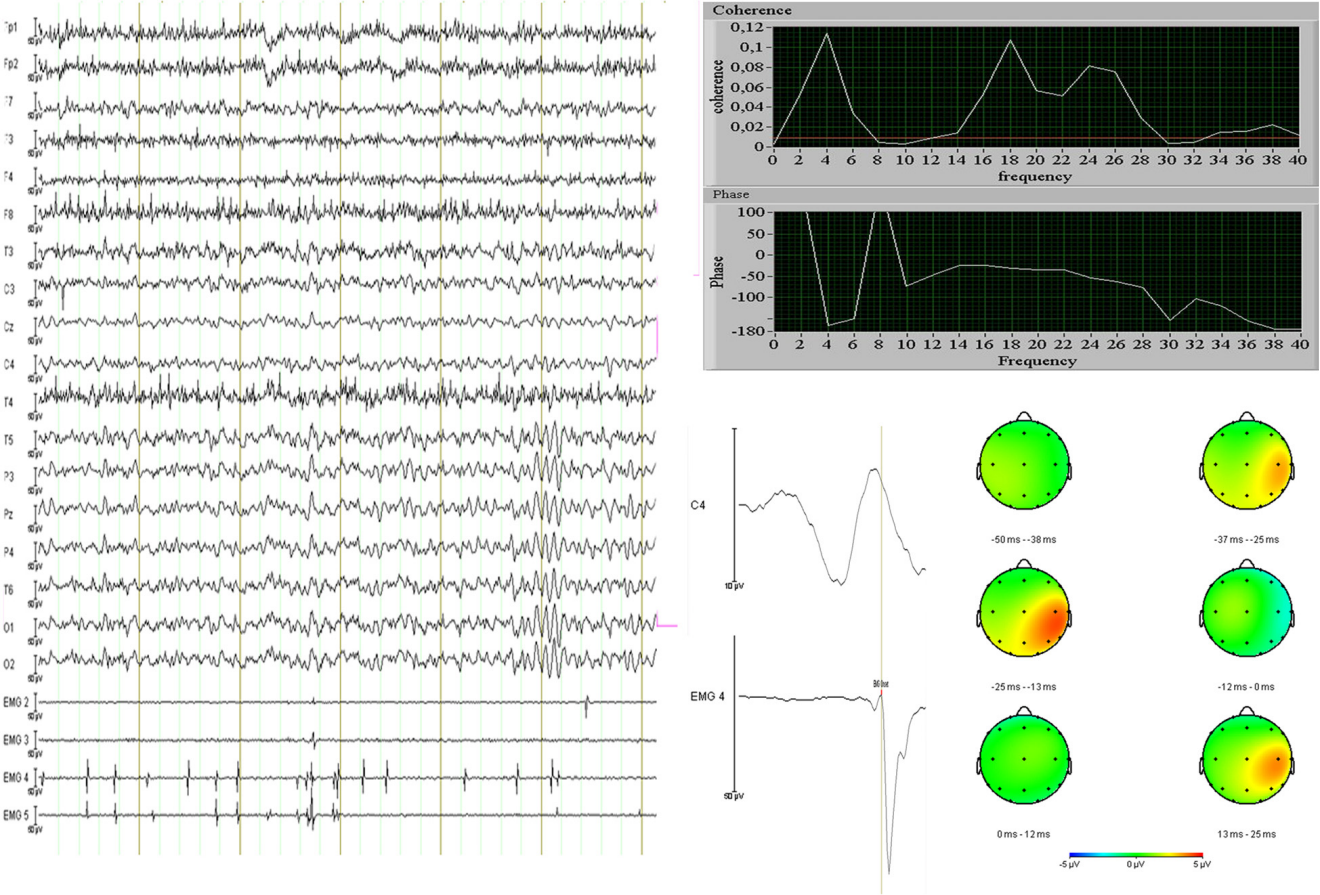
Electrophysiological characteristics of patient number 2. *Left Panel*: 6 s of raw EEG data with four left sided EMG channels, 2 and 3 are biceps and triceps, 4 and 5 are wrist flexor and extensor muscles. EEG montage: average reference. Note the multiple small myoclonic discharges, mostly in distal muscles, with small EMG burst duration and synchronous activation of agonists and antagonists. Background EEG pattern is normal without signs of epileptiform activity. *Right Panel*: coherence analysis (*upper panels*) shows significantly increased coherence from 14 to 28Hz, with also increased coherence around the myoclonus discharge frequency (around 4Hz). The *red line* is the threshold for significant coherence. The phase plot shows a linear decrease in phase from 14-28Hz (EEG leads EMG), with a calculated corticomuscular conduction time of 20,7 ms. The analysis is based on a segment of 180 s with frequently occurring myoclonus during rest. The *lower panels* show the result of back-averaging on the same segment. The average was based on 242 segments, with 75 ms pre-EMG onset and 25 ms post-EMG onset depicted. In the mapping view (view from above) a clear positive-negative potential field can be seen in the right centroparietal area. The positive peak is maximal at 23 ms before myoclonus onset

In all patients typical features of cortical myoclonus were found with short EMG burst duration, both negative and positive myoclonus types, and with a predominantly distal distribution. Activation of the myoclonus was induced by posture. A detailed description of patient 2 is seen in Fig 1.

### Cognitive dysfunction and psychiatric symptoms

Neuropsychological tests of five patients were available, in two patients new assessments were performed. In one patient no assessment was available. Not all patients have completed the same tests. Estimations of intelligence ranged from low average to average. Emotion recognition as a basic aspect of social cognition was compromised in the three assessed patients. Furthermore, patients showed impairments of working memory, attention, verbal fluency and learning of verbal information. Retention in long-term memory in relation to the imprinting was average to above average in three of our patients (Table 4).

**Table 4:** Results of neuropsychological assessment per domain

Domain		% Patients in 1st–5th percentile	% Patients in 6th percentile or above	Number of patients
Emotion recognition^a^	Total score	100	0	3
	% Patients in 1st–9th percentile	% Patients in 10th–49th percentile	% Patients in 50th percentile or above	
Intelligence^b^	29	43	28	7
Working memory^c^	57	43	0	7
Verbal learning^d^	86	14	0	7
Retention^d^	29	29	42	7
Attention^e^	80	20	0	5
Verbal fluency^f^	100	0	0	5

^a^Test used: FEEST, ^b^Tests used: WAIS-III, WAIS-IV, GIT-2, PPVT-III-NL, NART ^c^Tests used: WAIS-III digit span, WAIS-IV digit span, ^d^Test used: RAVLT, ^e^Test used: Trailmaking Test, ^f^Tests used: Fluency GIT-2, verbal fluency

Patients indicated elevated scores on the subscale somatic complaints of the Adult Self Report (ASR). Furthermore, patients and caregivers indicated elevated scores on the subscale attention problems. However, all scores were within the normal range and Wilcoxon signed rank tests showed no significant differences between patient and caregiver.

Three patients were affected by a psychotic disorder (nr. 1, 5, 8), one of them (nr. 1) was using antipsychotic medication during this study. Neuropsychological data were only available for two of these patients. Their scores were average compared to the other patients in the cohort.

### Quality of life and daily functioning

The health related quality of life in our cohort, measured with the RAND-36, was on several domains significantly lowered compared to the general population [24]. Especially the general health perception was scored remarkably low, 35.0 on a scale of 0–100. Other domains that were rated low were physical functioning, role limitations due to physical problems and vitality, while the domains social functioning, mental health and pain were on average relatively spared (Table 5).

**Table 5:** Health related quality of life

RAND-36 domains	Study population, mean (SD)	General population, mean (SD)	*p*-value*
Physical	43.1 (40.9)	81.9 (23.2)	*p* = 0.007
Social	73.4 (33.0)	86.9 (20.5)	*p* = 0.248
Role limitations (physical)	43.8 (37.5)	79.4 (35.5)	*p* = 0.008
Role limitations (emotional)	70.8 (45.2)	84.1 (32.3)	*p* = 0.406
Mental health	84.0 (10.5)	76.8 (18.4)	*p* = 0.055
Vitality	55.0 (16.5)	67.4 (19.9)	*p* = 0.035
Pain	93.1 (19.5)	79.5 (25.6)	*p* = 0.051
General health	35.0 (17.9)	72.7 (22.7)	*p* < 0.001
Health change	50.0 (29.9)	52.4 (19.4)	*p* = 0.821

*SD* standard deviation

*Independent samples *t*-test for equality of means, significance level *p* < 0,05

The level of functional disability in daily life, expressed by the functional disability index (FDI, range 0–3), in our patients was 1.56 (SD 1.02). This is considerably higher than reference values of the general population, where mean values of 0.03 to 0.05 are reported [23] (Mann-Whitney *U* test: *p* = 0.001), indicating more functional disabilities in our cohort.

It appeared that patients with a more severe movement disorder as measured with the GCI Scale had a significantly higher FDI (Spearman’s Rho: rs = 0.814, *p* = 0.014). Also with another measure of impact on daily life, the SIP-68, a significant relation was found between the severity of the movement disorder and somatic autonomy (Spearman’s Rho: rs = 0.706, *p* = 0.050) and physiological autonomy/communication (Spearman’s Rho: *r* =0.727, *p* = 0.041).

The RAND-36 domain concerning physical functioning was correlated with the severity of the movement disorder; patients with more severe movement disorders experienced more physical disabilities (Spearman’s Rho: rs = −0.845, *p* = 0.008). No significant relation was shown for the other QoL domains including emotional functioning and well-being, energy, social functioning, pain, general health and health change.

## Discussion

We systematically assessed the presenting and current symptoms in NP-C, with a special focus on movement disorders and neuropsychiatric functioning. Not unexpectedly, the most common movement disorder was ataxia, but myoclonus was seen in five out of eight patients. Furthermore dystonia was frequently seen and one patient showed a tic. Neurological signs are very common in NP-C and movement disorders represent an important part of the neurological phenotype. Although the presence of movement disorders was not an inclusion criterion, all included patients in our cohort were routinely seen by a neurologist, implicating a potential selection bias. However, according to the literature only a minority (13 %) of the patients with NP-C do not display any neurological manifestations at all and most of these patients have the infantile form of NP-C [2].

In all our patients movement disorders were a presenting neurological feature, often in combination with VSGP, psychosis or cognitive deficits. Movement disorders and psychosis were reason to seek medical attention; VSGP and cognitive decline were not. However, also in patients with movement disorders help-seeking delay was found, although the exact time span in our cohort is not known. Reason for this is that only the moment of first symptoms and final diagnosis were reported in the medical history, data about previous visits to general practitioners and other hospitals before referral to an Academic Center were not available.

Incidence of ataxia as first presenting symptom in literature is 20 %, other movement disorders cover 11 % [5]. This is considerably lower than in our cohort. Reason for this might be the higher age of onset of NP-C in our study. Movement disorders and psychiatric symptoms are the most common presenting symptoms in adolescence and adult NP-C [1]. A combination of two or more movement disorders, in this age group present in seven patients, should raise suspicion of NP-C, especially in combination with eye movement disorders or cognitive deficits.

We found myoclonus in five out of eight patients, of which in three of them it was the first presenting movement disorder. The reported frequency of myoclonus in NP-C in the literature is low, but our study suggests that this may be higher than expected. It is not unlikely that myoclonus in NP-C is not always recognized and we can imagine that when myoclonus is more subtle and multiple movement disorders are present, this can be overlooked. Especially in patients with other movement disorders that may be more dominant, subtle jerks can be easily missed [10]. Recognition of myoclonus is very important. Firstly because having an extra movement disorder can be an important clue for diagnosing NP-C. Furthermore, myoclonic jerks can be very disabling and symptomatic treatment is possible. EEG and polymyography showed that the myoclonus in NP-C has a cortical origin and that it consists of both positive and negative myoclonus. Furthermore, both low frequency and high frequency myoclonus were found. Previous reports in two patients also detected a cortical myoclonus [4, 9]. For the treatment of cortical myoclonus, levetiracetam or piracetam are considered drugs of first choice. Alternatives are clonazepam and valproic acid [25, 26]. The patients in our cohort experienced significantly more functional disabilities compared to healthy controls and a significant relation was found between the severity of the movement disorder and psychical disabilities. Recognition and adequate treatment of myoclonus are likely to positively influence daily functioning, but further treatment studies are required to support this hypothesis.

Except one, all patients showing myoclonus also had cerebellar ataxia. Cortical myoclonus is thought to result from increased excitability of the sensorimotor cortex due to decreased inhibition through the cerebellar-thalamic-cortical loop [27]. The combination of cortical myoclonus and cerebella ataxia is also seen in disorders with prominent Purkinje cell loss, for example celiac disease [28, 29]. In NP-C brains, ballooned neurons, axonal abnormalities, neurodegeneration and astroglyosis have been described. Especially Purkinje cells of the cerebellum are vulnerable to early damage and cell loss begins early in disease [30, 31]. Damage to Purkinje cells may play a role in cortical myoclonus and ataxia in NP-C and these pathological findings support the shared pathology of cortical myoclonus and ataxia in our patients. With loss of Purkinje cells being a prominent feature of NP-C and not only playing a role in ataxia but in myoclonus as well, this also suggest that incidence of myoclonus in NP-C might be higher than currently estimated. The clinical syndrome of myoclonus and ataxia in combination of relatively mild epilepsy and mental retardation is called progressive myoclonus ataxia (PMA) or the Ramsay Hunt syndrome, [32] and seems applicable in many cases with NP-C.

Present in seven patients, ataxia was the most common movement disorder in our group and is underscoring previous reports [2, 8]. However, it is important to mention that not in all NP-C patients ataxia was the presenting movement disorder. In three patients ataxia developed later during the course of the disease. Hence, ataxia was not the most prominent movement disorder in all patients. In three patients with ataxia, myoclonus was more severe. So, ataxia is an important neurological hallmark of NP-C, but its absence does not exclude NP-C.

Besides movement disorders, cognitive deficits and psychiatric features are early symptoms of NP-C, especially in the adolescent and adult form [5]. Two patients developed a psychosis early in disease and also cognitive deficits were among the early symptoms. Patients in our sample showed cognitive deficits in all domains investigated, in particular in working and verbal memory and attention. This confirms earlier findings by Klarner et al. [16]. We focused on social cognition in three patients and found deficits in emotion recognition. More studies with bigger sample sizes are needed in order to clarify whether these deficits in social cognition are typically for NP-C and when they develop in the course of the disease. This might help in identifying patients with NP-C presenting with cognitive problems early in the course of the disease and discriminating them from patients with other neuropsychiatric disorders.

## Conclusion

We presented evidence that movement disorders, psychiatric symptoms and cognitive deficits are important features of NP-C. Cortical myoclonus was seen in more than half of the patients. The role of myoclonus in NP-C is not yet established, but our study suggests that incidence may be higher than considered until now. Careful examination is required to detect myoclonus and other movement disorders as they sometimes require specific treatment. Nowadays, diagnosing NP-C is still a challenge for physicians. In adolescent and adult patients presenting with mostly psychiatric and neurological signs, knowledge of the exact phenotype of these symptoms is very important in order to diminish diagnosis delay. Further research is needed to determine the exact role of myoclonus in NP-C.

## Abbreviations

EEG- EMG: Electroencephalography- electromyography
GCI: Global Clinical Impressions Scale
HRQol: Health related quality of life
NP-C: Niemann-Pick type C
VSGP: Vertical supranuclear gaze palsy
